# Performance Research and Engineering Application of Fiber-Reinforced Lightweight Aggregate Concrete

**DOI:** 10.3390/ma17225530

**Published:** 2024-11-13

**Authors:** Feifei Jiang, Wencong Deng, Qi Wang, Jialei Wang, Zhongyang Mao

**Affiliations:** 1 School of Civil Engineering, Nantong Institute of Technology, Nantong 226000, China; 999620140019@just.edu.cn; 2 China State Construction Engineering (Macau) Co., Ltd., Macau 999078, China; deng_wencong@163.com (W.D.); wangq202312@163.com (Q.W.); 3 College of Materials Science and Engineering, Nanjing Tech University, Nanjing 211800, China; mzy@njtech.edu.cn

**Keywords:** fiber, lightweight aggregate concrete, mechanical properties, microstructure, freeze–thaw

## Abstract

Low strength and low impact toughness are two of the main issues affecting the use of lightweight aggregate concrete in harsh cold environments. In this study, the strength of concrete was improved by adding high-strength fibers to bear tensile stress and organize crack propagation. Four sets of comparative experiments were designed with freeze–thaw cycles of 0, 50, 100, and 150 to study the mechanical properties of fiber-reinforced lightweight aggregate concrete under freeze–thaw conditions. A detailed study was conducted on the effects of freeze–thaw on the compressive strength, flexural strength, impact toughness, and microstructure of concrete with different fiber contents (3, 6, and 9 kg/m^3^). The results show that for ordinary lightweight aggregate concrete, under the freeze–thaw cycle, the internal pore water of the concrete froze and generated expansion stress, resulting in tensile cracks inside the concrete. The cracks gradually accumulated and expanded, ultimately leading to cracking and damage of concrete structures. After 150 cycles, the strength loss rate exceeded 25%. When adding a reasonable amount of fiber (6 kg/m^3^), the fiber took on the tensile stress and hindered the development of internal cracks, significantly enhancing the splitting tensile strength, flexural strength, and impact toughness of lightweight aggregate concrete. And the failure pattern of concrete was significantly improved. At the beginning of the freeze–thaw cycle, the internal tensile stress was less than the fiber tensile strength and the fiber–matrix bonding strength, and the strength reduction rate of the concrete was slow. Relying on the friction absorption capacity between the fiber and the matrix, the fiber used its own deformation to resist the tensile stress. In the late stage of the freeze–thaw cycle, due to the destruction of the fiber–matrix transition zone structure, the bond strength decreased, the crack resistance and toughening effect decreased, and the strength of the concrete decreased rapidly. Moreover, the reduction in impact toughness was greater than the compressive strength and flexural strength under static load.

## 1. Introduction

In cold regions, building materials often face freeze–thaw cycles, leading to gradual deterioration of material properties and seriously affecting the service life and safety of structures [1,2]. As one of the most widely used building materials, concrete’s frost resistance directly affects the durability of engineering structures [2,3,4]. However, traditional concrete has many internal pores and is prone to water absorption. After multiple freeze–thaw cycles, it is prone to cracking, which in turn affects its mechanical properties [5,6]. In recent years, lightweight aggregate concrete has been widely used in construction projects due to its light weight, good thermal insulation performance, and good frost resistance [7,8,9,10].

Lightweight aggregate concrete is a concrete material made by replacing ordinary aggregates with lightweight aggregates (such as ceramic particles, expanded shale, etc.), which can effectively reduce the weight of structures and is widely used in construction projects, especially in super-high-rise buildings and cold regions [11,12,13,14]. Yoon has found that replacing natural stones with lightweight aggregates reduces the self-weight by 25–30%. This not only reduces the requirement for the bearing capacity of beams and columns, but also reduces the size of foundations and supporting components, reduces the use of steel and concrete, and ultimately achieves the goals of weight reduction and material conservation [15]. However, lightweight aggregate concrete also faces the challenge of freeze–thaw cycles, and its mechanical performance changes under multiple freeze–thaw cycles have not been fully studied. Moreover, Safwan has found that due to its porous nature, lightweight aggregate has a lower strength, and the aggregate is prone to cracking when subjected to tensile and shear forces. Once it cracks, the low strength of the aggregate makes it difficult to prevent crack propagation, resulting in a lower residual strength of the concrete after cracking [16]. Therefore, how to improve the strength of lightweight aggregate concrete is currently a research hotspot. Li [17] carried out rapid freeze–thaw tests on concrete specimens and analyzed the damage evolution law of concrete under uniaxial compression with different freeze–thaw damage degrees. It was found that the compression failure of specimens after freeze–thaw damage showed more obvious brittle characteristics. Therefore, how to improve the strength of lightweight aggregate concrete and improve the toughness after freeze–thaw damage is the current research hotspot.

Fiber-reinforcement technology is widely used in the research and engineering practice of concrete to improve its strength and toughness. Fibers can not only effectively improve the tensile strength and crack resistance of concrete, but also enhance its toughness and impact resistance. Guler’s research found that adding 0.75% fibers increased the compressive strength, tensile strength, and flexural strength of concrete by 1.1%, 73.4%, and 47.9%, respectively [18]. However, freeze–thaw cycles can damage the bond strength between fibers and the matrix, reducing the performance of fiber-reinforced concrete [19,20,21]. Especially in cold regions, frequent freeze–thaw cycles can cause the expansion of microcracks inside concrete and the deterioration of the material structure, thereby reducing its bearing capacity and service life. Zeng found that after 150 freeze–thaw cycles, the strength of fiber-reinforced concrete decreased by 17–38% [22]. How to improve the frost resistance of fiber-reinforced concrete is currently a key issue restricting its promotion in cold regions of China. Dong [23] found that micropores in lightweight aggregate can provide “buffer space” during freeze–thaw cycles. These pores help to reduce the concentration of freeze–thaw stress and prevent cracks in concrete. In addition, lightweight aggregate concrete can absorb and store water, provide more uniform ice crystal distribution at low temperatures, reduce internal stress, and help to avoid water concentration in the same place. Therefore, lightweight aggregate can improve the frost resistance of concrete.

Through the above analysis, the replacement of natural aggregate with lightweight aggregate can reduce the self-weight of the structure and improve the frost resistance of concrete. Fiber can improve the toughness and strength of lightweight aggregate concrete. Considering the natural conditions of cold winter in cold regions of China, and the adverse factors of a lack of natural aggregate, this paper uses lightweight aggregate and plastic steel fiber to prepare fiber-reinforced lightweight aggregate concrete. Compared with steel fibers and basalt fibers commonly used in engineering, plastic steel fibers have the advantages of a large elastic modulus, high tensile strength, and non-rusting [24]. The effect of fiber on the mechanical properties and microstructure of lightweight aggregate concrete under freeze–thaw cycles was studied in detail. The research results are conducive to improving the durability of concrete structures in severe cold regions.

## 2. Materials and Methods

### 2.1. Materials

Cement: PO 42.5 ordinary Portland cement. The main chemical composition of the cement is shown in Table 1. Coarse aggregate: spherical fly ash ceramsite produced by Baotou Jingzheng Ceramsite Co., Ltd. (Baotou, China), with a particle size of 5–20 mm, a bulk density of 0.97 g/cm^3^, an apparent density of 1.76 g/cm^3^, and a water absorption rate of 12.25%. Fine aggregate: river sand, bulk density was 1.58 g/cm^3^, apparent density was 2.46 g/cm^3^. Fiber: High-performance special-shaped plastic steel fiber produced by Zhejiang Ningbo Dacheng New Materials Co., Ltd. (Ningbo, China). The diameter was 1.2 mm, the length was 30 mm, the density was 0.97 g/cm^3^, the elastic modulus was 9884 MPa, the tensile strength was 543 MPa, and the elongation was 14.1%. Water reducer: naphthalene-based B2 high-efficiency water reducer produced by Jiangsu Subote New Materials Co., Ltd. (Nanjing, China), with a water reduction rate of 20%.

### 2.2. Methods

**Figure 1:**
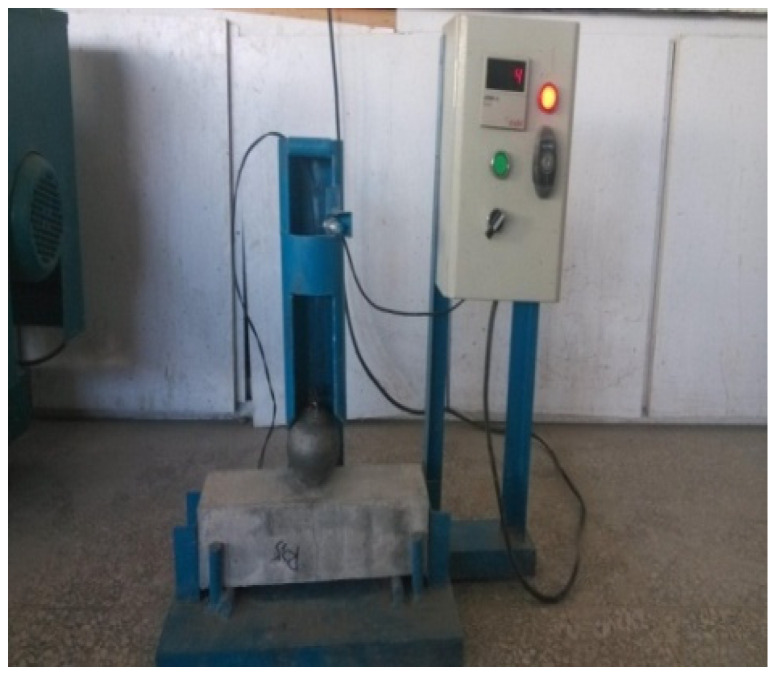
Impact test equipment for fiber-reinforced lightweight aggregate concrete.

(1) The mix proportion of fiber-reinforced lightweight aggregate concrete was designed with reference to the loose volume method in the Technical Specification for Lightweight Aggregate Concrete (JGJ51-2002) [25]. The specific mix proportions are shown in Table 2. Considering the strength and workability requirements of concrete, the fiber content was initially set to 0 kg/m^3^, 3 kg/m^3^, 6 kg/m^3^, and 9 kg/m^3^, respectively.
(2) Previous studies have shown that as the fiber content increases, the fibers tend to clump and become difficult to stir. The experiment used a small, forced concrete mixer for mixing, with mixing performed in stages. The lightweight aggregate was pre-wetted for 1 h before mixing. After the specimens were formed, they were cured in a standard curing room for 28 days before being taken out for freeze–thaw testing.
(3) In order to study the effect of freeze–thaw cycles on the mechanical properties of fiber-reinforced lightweight aggregate concrete, comparative tests were conducted with freeze–thaw cycles of 0, 50, 100, and 150 times, respectively, while keeping other test environments and material components unchanged. Compressive and flexural tests of fiber-reinforced lightweight aggregate concrete under different freeze–thaw cycles were carried out to study the influence of freeze–thaw cycles on the mechanical strength of fiber-reinforced lightweight aggregate concrete. The freeze–thaw test was conducted following the “Standard for Test Methods of Long-Term and Durability Performance of Ordinary Concrete” (GB/T50082-2009) [26]. After being cast, the concrete was cured for 24 days, then soaked in water at a temperature of (20 ± 2) °C for 4 days before the freeze–thaw testing began. Each freeze–thaw cycle was completed within 4 h, with the specimen’s temperature controlled at a minimum of −17 ± 2 °C and a maximum of 5 ± 2 °C.
(4) Impact toughness can characterize the ability of concrete materials to absorb energy under dynamic loads. The higher the impact toughness of concrete, the stronger its ability to resist dynamic loads, and vice versa. The impact resistance test of fiber-reinforced lightweight aggregate concrete was carried out according to the concrete impact resistance test method, and the effects of the freeze–thaw cycles and fiber content on the impact toughness of lightweight aggregate concrete were analyzed. The test used a drop hammer impact tester, as shown in Figure 1, with a drop hammer mass of 3 kg and an impact height of 300 mm.
(5) A scanning electron microscope (SU3500, HITACHI, Tokyo, Japan) was used to scan the fiber–matrix interface transition zone. The effects of freeze–thaw cycles on the interface transition zone were compared to analyze the internal mechanism of changes in the macroscopic properties of concrete.

## 3. Results and Discussion

### 3.1. Effect of Fiber Content on Compressive Strength

**Figure 2:**
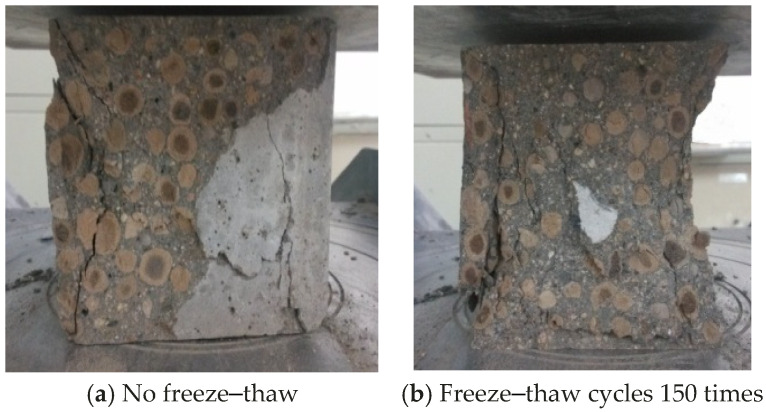
Compressive failure form of ordinary lightweight aggregate concrete.

The compression failure morphology of ordinary lightweight aggregate concrete is shown in Figure 2. There was a hoop effect between the test block and the pressure plate of the press, and the failure was manifested as two conical failures at the upper and lower apex angles. From the failure section, most of the cracks passed through the aggregate, the lightweight aggregate itself was damaged, and the stress on the aggregate reached its strength limit. It was very different from ordinary concrete under compression. When ordinary concrete was compressed and damaged, the aggregate was not damaged due to its high strength. The damage occurred in the weaker matrix and interface. This shows that the self-strength of the lightweight aggregate had a great influence on the compressive strength of the fiber-reinforced lightweight aggregate concrete. The strength was further reduced after freezing and thawing, and large pieces of concrete peeled off on the side.

**Figure 3:**
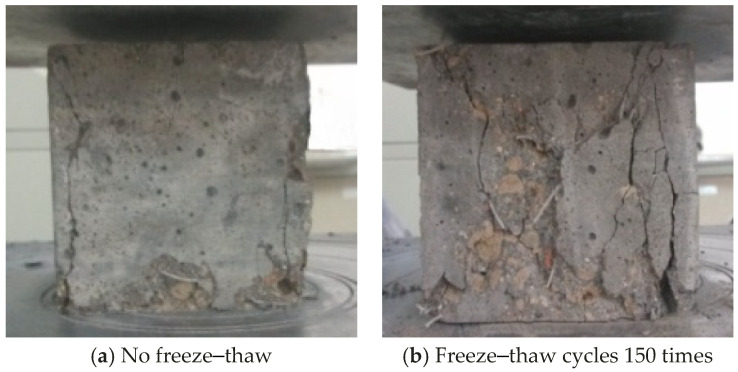
Failure form of fiber-reinforced lightweight aggregate concrete under compression.

It can be seen from Figure 3 that after adding fibers, the surface of the unfrozen–thawed test block was smooth, and there was no exposure of fibers and aggregates. There was a crack at the damaged area with a larger width, but no large-scale whole-piece falling off occurred. Due to the restraining effect of the fibers on the cracks, the fiber-reinforced lightweight aggregate concrete test block showed a certain ductility when it was destroyed, and the integrity was good after destruction. The surface of the test block after the freeze–thaw cycle was rough and uneven, some fibers and aggregates were exposed, and there was a whole-piece falling off on the side. This is mainly due to the fact that after multiple freeze–thaw cycles, frost heave cracks were generated inside the test block, and there were large initial defects [27,28]. After being compressed, the internal cracks further expanded and gradually penetrated to the surface, causing overall peeling.

**Figure 4:**
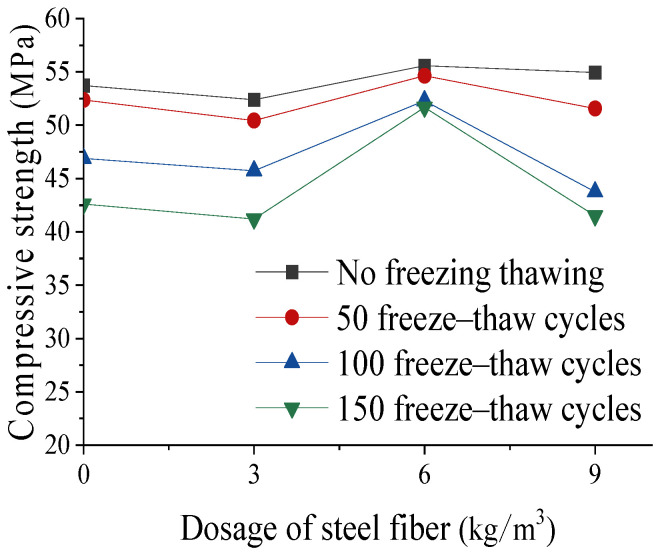
Effect of fiber content on compressive strength.

Figure 4 shows the measured compressive strength of the concrete. The strength of the concrete showed a trend of first decreasing, then increasing, and then decreasing again with the increase in the fiber content. After freeze–thaw, the strength of each specimen decreased to varying degrees. The main reasons may be as follows: (1) Concrete with a content of 3 kg/m^3^ has too few fibers, which cannot effectively bear the tensile stress caused by compression and have little effect on crack limitation. At the same time, the addition of fibers introduces a weak interface between the fibers and matrix, which is not as dense as ordinary concrete structures, resulting in lower strength compared to ordinary concrete [29]. (2) When the dosage is 9 kg/m^3^, due to too many fibers, the weak surface of the fibers in contact with the cement matrix increases, and the fibers are not fully wrapped by the cement slurry, resulting in more defects and a decrease in the bond strength between the fibers and the cement slurry, leading to a decrease in the concrete strength [30]. (3) The concrete fiber content with a dosage of 6 kg/m^3^ is moderate, and the strength is significantly improved. The fibers suppress the development of cracks without introducing too much of a weak interface with the mortar.

### 3.2. Effect of Freeze–Thaw Cycles on Compressive Strength

**Figure 5:**
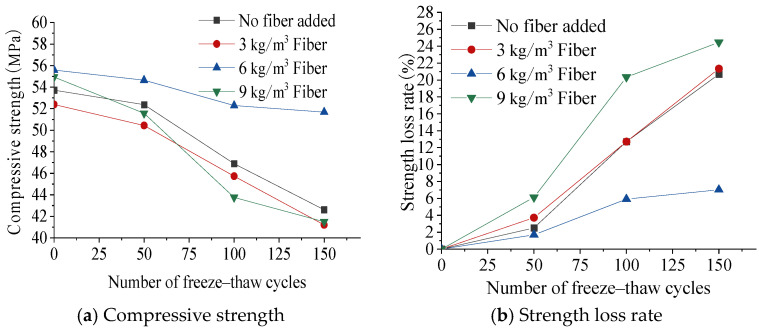
Effect of freeze–thaw cycles on concrete compressive strength and strength loss rate.

The effects of freeze–thaw cycles on the compressive strength and strength loss rate are shown in Figure 5. As the number of freeze–thaw cycles continued to increase, the concrete strength loss rate continued to increase, and the compressive strength gradually decreased. In the early stage of the freeze–thaw cycle (0 to 50 times), the compressive strength decreased slowly, and the strength loss rate was controlled within 2–6%. In the late stage of the freeze–thaw cycles (100 to 150 times), the compressive strength decreased rapidly, and the strength loss rate could reach as high as 24.5%. This is because during the freeze–thaw cycle, the pore water inside the concrete freezes and produces expansion stress, resulting in tensile cracks inside the concrete. Under the action of repeated freeze–thaw cycles, the cracks gradually accumulate and expand, eventually leading to the destruction of the concrete structure and a reduction in the compressive strength.

As can be seen from Figure 5b, the compressive strength loss rate of the lightweight aggregate concrete with a fiber content of 6 kg/m^3^ was the smallest, which was 13.7% lower than that of ordinary concrete. The strength reduction rate was significantly lower than that of the other fiber-reinforced lightweight aggregate concretes. This was because the reasonable fiber content enabled the slurry to fully wrap the fibers, resulting in a high fiber–matrix interface bonding strength, a dense structure, and few defects.

### 3.3. Effect of Freeze–Thaw Cycles on Splitting Tensile Strength

**Figure 6:**
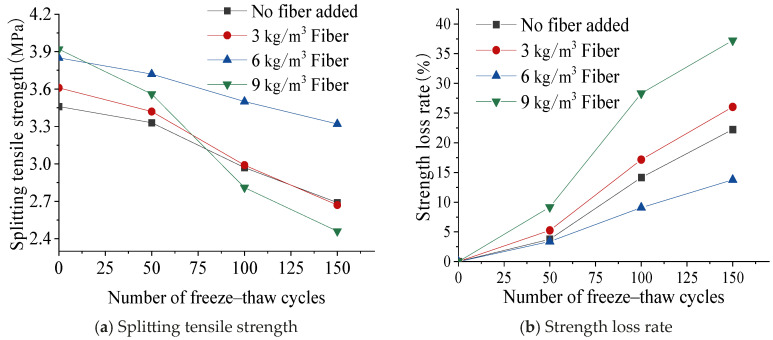
Effect of freeze–thaw cycles on splitting tensile strength and strength loss rate.

Figure 6 shows the influence of freeze–thaw cycles on the splitting tensile strength. When the fiber content was low (0 kg/m^3^, 3 kg/m^3^, and 6 kg/m^3^), the splitting tensile strength of the concrete decreased relatively slowly in the early stage of freeze–thaw, and gradually accelerated with the increase in freeze–thaw cycles in the later stage. When the fiber content increased to 9 kg/m^3^, the loss rate of the splitting tensile strength was much higher than that of other concretes. This is because adding too much fiber increased the interface between the fiber and the cement matrix, and the damage to the interface area caused by freeze–thaw action also increased, resulting in a decrease in the bond strength between the fiber and the matrix. When the concrete was subjected to splitting load, the fiber with a low bond strength with the matrix would not be able to effectively disperse the stress concentration at the tip of the crack, and the fiber that can continue to bear tensile stress was greatly reduced, thereby reducing the splitting tensile strength of the concrete. The concrete with a fiber content of 6 kg/m^3^ had the smallest strength loss rate due to its reasonable content.

### 3.4. Effect of Freeze–Thaw Cycles on Flexural Strength

**Figure 7:**
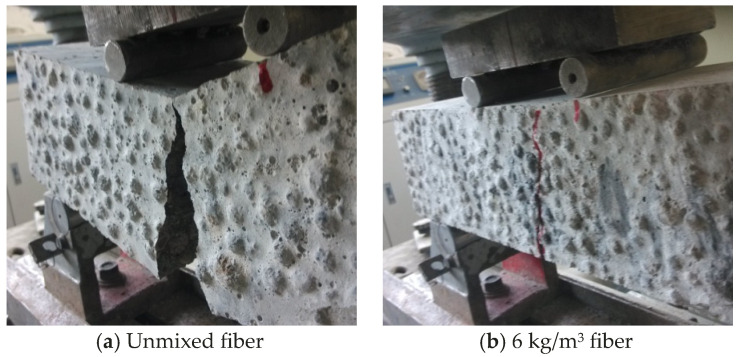
Flexural failure morphology of lightweight aggregate concrete.

Flexural strength is an important indicator of concrete’s tensile properties. The fiber-reinforced lightweight aggregate concrete was subjected to flexural tests using the three-point loading method, and the failure morphology is shown in Figure 7. Comparison with Figure 7 shows that the lightweight aggregate concrete without fibers fractured brittlely under load. When the first main crack appeared on the lower surface of the specimen, the cracks rapidly expanded upward, causing the specimen to break suddenly. Lightweight aggregate concrete with 6 kg/m^3^ fiber showed good ductility characteristics under load. When cracks appeared on the surface of the specimen, the cracks slowly extended with the increase in load. When the specimen was destroyed, the crack width was small due to the constraint of the fiber, and the cracking changed from a main crack to parallel fine cracks. Finally, the specimen remained as a whole when it was destroyed.

**Figure 8:**
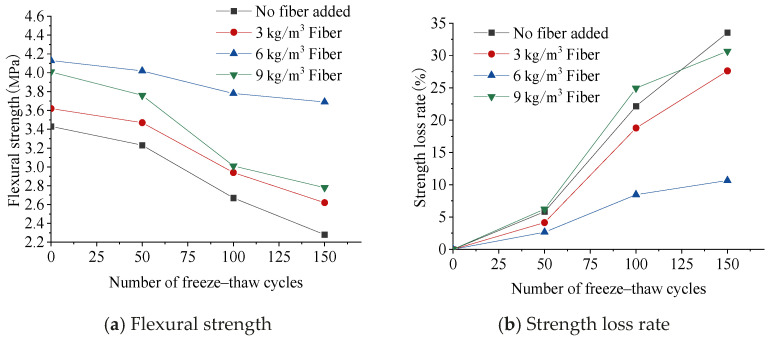
Effect of freeze–thaw cycles on flexural strength.

Figure 8 shows the change in flexural strength. As can be seen from Figure 8a, the flexural strength of the concrete gradually decreased with the increase in the number of freeze–thaw cycles. The rate of decrease was slow in the early stage of the freeze–thaw cycle; the flexural strength decreased rapidly in the later stage. Because the elastic modulus of the fiber was much smaller than that of the concrete matrix, the fiber and the matrix could not deform synchronously. When the load exceeded the tensile strength of the matrix, the matrix cracked and partially stopped working. This part of the load was borne by the fiber. At this time, the concrete relied on the tensile strength of the fiber itself and the friction between the fiber and the matrix to consume energy at the same time. When the tensile stress was higher than the bonding strength between the fiber and matrix, the fiber would slip or even pull out. According to the above mechanism, the bonding strength between the fiber and the matrix had a great influence on the overall flexural strength of the concrete, and repeated freeze–thaw cycles greatly weakened the bonding strength, resulting in a decrease in the flexural strength.

As can be seen from Figure 8b, with the increase in freeze–thaw cycles, the flexural strength loss rate of lightweight aggregate concrete with a fiber content of 6 kg/m^3^ is 22.9% lower than that of ordinary concrete and was also much lower than that of concrete with a content of 3 kg/m^3^ and 9 kg/m^3^. This was because under repeated freeze–thaw cycles, a large fiber content would cause the interface defects to gradually increase with the accumulation of freeze–thaw damage, while fewer fibers could not effectively share the tensile stress. In addition, freeze–thaw cycles would greatly reduce the bonding strength between the fiber and the matrix, leading to accelerated damage to the flexural strength of fiber-reinforced lightweight aggregate concrete.

### 3.5. Effect of Freeze–Thaw Cycles on Impact Toughness

**Figure 9:**
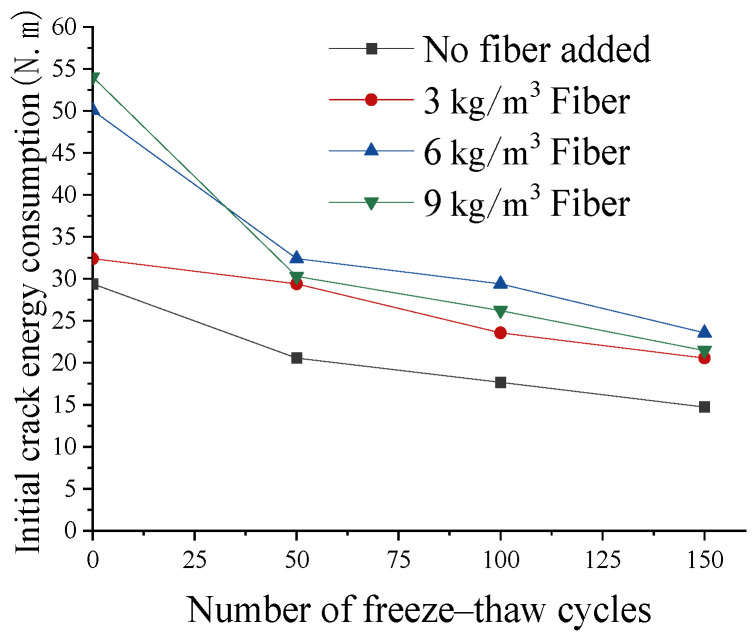
Effect of freeze–thaw cycle on initial crack energy consumption.

The effect of freeze–thaw cycles on the initial crack energy consumption is shown in Figure 9. The initial crack energy consumption of lightweight aggregate concrete without freeze–thaw cycles increased with the increase in the fiber content, mainly because the initial crack in the concrete was related to the number of fibers distributed horizontally in the bottom tensile zone, that is, the bottom fibers could effectively offset part of the impact energy consumption [31]. With the increase in freeze–thaw cycles, the impact toughness of the fiber-reinforced coarse aggregate concrete decreased rapidly, and the influence of freeze–thaw damage on the impact toughness was greater than the compressive strength and flexural strength under static load. Before freeze–thaw, the initial crack energy of the fiber concrete (6 kg/m^3^) was 76% greater than that of ordinary concrete, but after the cycle, it was only 49% greater. This was mainly because the freeze–thaw cycle destroyed the bonding strength of the fiber–matrix interface and reduced the ability of the fiber to withstand impact loads.

**Figure 10:**
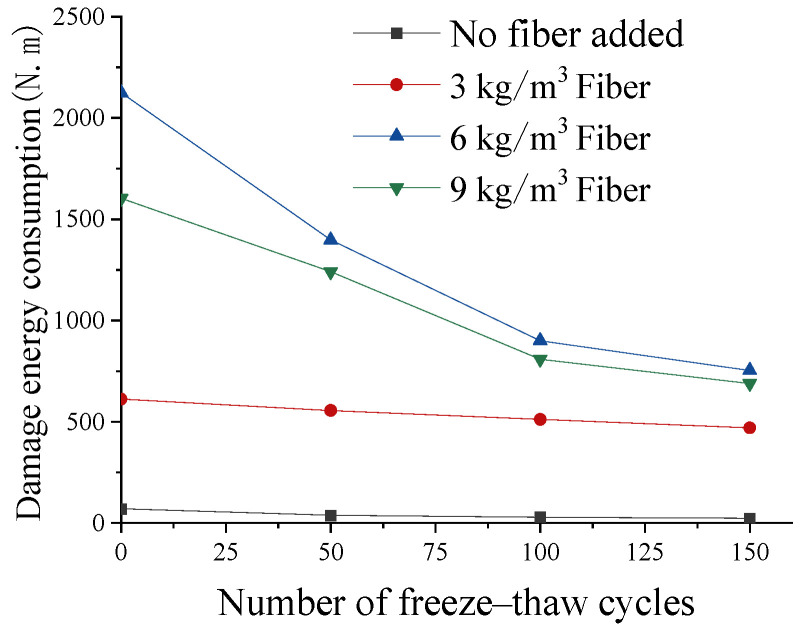
Effect of freeze–thaw cycles on damage energy consumption.

The destruction energy consumption of different specimens is shown in Figure 10. The damage energy consumption of lightweight aggregate concrete increased with the increase in fiber content. The addition of fiber significantly improved the energy consumption ability of concrete to resist damage. The damage energy consumption of lightweight aggregate concrete without fiber and with a fiber content of 3 kg/m^3^ is much lower than that of lightweight aggregate concrete with a fiber content of 6 kg/m^3^ and 9 kg/m^3^. With the increase in freeze–thaw cycles, the destructive energy consumption gradually decreased. Freeze–thaw had a greater weakening effect on the destructive energy consumption of concrete with a higher fiber content. However, lightweight aggregate concrete with fiber added still had a stronger ability to offset the impact energy consumption after freeze–thaw cycles than that without fiber.

### 3.6. Microstructure of Fiber–Matrix Interface Transition Zone

**Figure 11:**
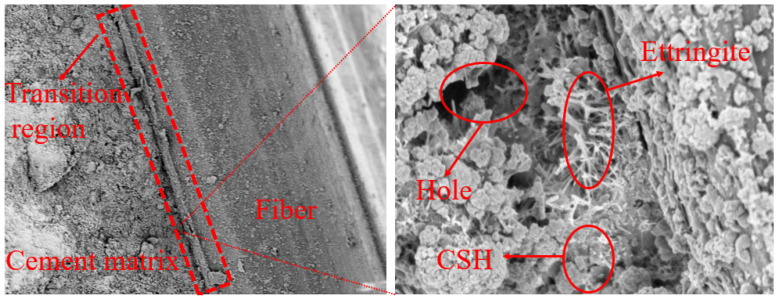
Microstructure of the transition zone between fibers and matrix interface before freeze–thaw cycles.

The fiber–matrix interface of fiber-reinforced lightweight aggregate concrete before freeze–thaw is shown in Figure 11. There was a clear boundary between the fiber and the matrix, some pores existed in the interface transition zone, many coarse needle-like and columnar calcium aluminates spanned between the C-S-H gels, and a large number of gelled particles and some coarse crystals accumulated on the fiber surface. The fibers were different from lightweight aggregates, as the surface of the fibers was hydrophobic. Due to the micro-bleeding effect, water was concentrated on the fiber surface, which resulted in a thicker water film layer. This reduced the ion concentration and reduced the formation of hydration products on the fiber surface, affecting the bonding strength between the fiber and cement matrix. The fiber–matrix interface transition zone is mainly composed of three parts. The first part is a double-layer membrane, which mainly contains Ca(OH)_2_ crystals and hydrated calcium silicate gel. The second part is the Ca(OH)_2_-rich area. There are some pores inside this part, which make the structure not dense, resulting in a weak bond between the fiber and the matrix. The third part is the porous area, which has a large porosity and is another weak link in the interface layer. Therefore, when the fiber content is too large (9 kg/m^3^), a large number of weak fiber–matrix interfaces were brought into the composite system, which reduced the strength.

**Figure 12:**
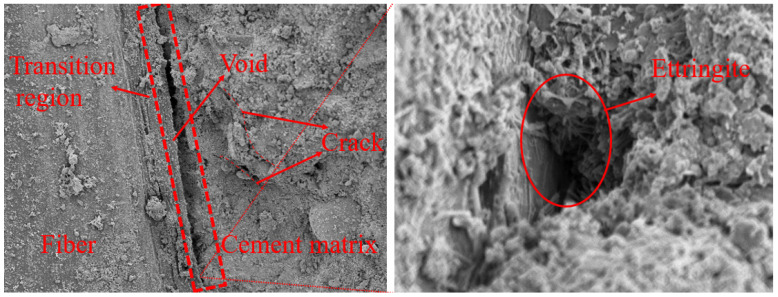
Microstructure of the transition zone between fibers and matrix interface after freeze–thaw cycles.

The fiber–matrix interface of fiber-reinforced lightweight aggregate concrete after 150 freeze–thaw cycles is shown in Figure 12. Due to freeze–thaw, ettringite and CSH gel broke and separated in the fiber–matrix interface area, resulting in wider gaps, which reduced the integrity of the fiber–matrix interface and ultimately resulted in the fiber being unable to effectively exert its reinforcement, toughening, and crack resistance capabilities. Therefore, the tensile strength, flexural strength, and impact toughness were reduced due to the effects of freeze–thaw. According to osmotic pressure theory, the solution in the macropores in the transition zone froze first, and the ion concentration in the solution increased, forming a concentration difference with the nearby small pores of the cement matrix. Under the action of this concentration difference, the pore solution in the smaller pores in the matrix migrated to the macropores in the interface transition zone, thereby generating osmotic pressure, which led to the partial rupture of the columnar calcium aluminate and CSH gel that acted as a bridge in the interface transition zone, resulting in a decrease in the bonding strength between the fiber and the matrix, and ultimately leading to the attenuation of the mechanical properties of the concrete. At the same time, due to the inherent microcracks in the concrete caused by freezing and thawing, the initial strength of the concrete was reduced. In the subsequent load test, the cracks would further expand under the load and cause structural damage. Therefore, the performance of concrete is influenced by both the fiber content and freeze–thaw cycles. The decline rate of the compressive strength, splitting compressive strength, and flexural strength of concrete with a different fiber content and different freeze–thaw time are shown in Table 3.

Through the above analysis and comparison with the literature [32], it can be found that the freeze–thaw damage of fiber-reinforced lightweight aggregate concrete is obviously different from that of ordinary concrete. During freezing and thawing, the water in the pores of ordinary concrete freezes and expands, forming an ice wedge effect, which leads to the continuous expansion of pores and cracks, reducing the strength of the matrix. For fiber-reinforced lightweight aggregate concrete, freeze–thaw not only affects the strength of the matrix, but also leads to the deterioration of the microstructure of the fiber–matrix transition zone, which directly leads to the reduction in the bond force between the fiber and the matrix. With the increase in freeze–thaw cycles, the “bridging” effect of the fiber continues to weaken, and the fiber is difficult to effectively prevent the expansion of cracks, resulting in a significant decline in the tensile strength and flexural strength of the concrete. The damage of these microstructures can well explain why the macro mechanical properties are reduced. These macroscopic performance deteriorations are directly related to the damage of the microstructure in the transition zone.

### 3.7. Engineering Application

The construction project was a super-high-rise building in China. Six 149.90 m residential buildings with 50 floors and a 1-floor basement were built in the project. The project covered an area of about 20,000 m^2^ and a building area of about 330,000 m^2.^ The project was located in the reclamation area, with loose soil and a low bearing capacity, so the project adopted a high-strength prestressed concrete pipe pile foundation, with a diameter of 600 mm and a depth of about 43 m. At the same time, in order to reduce the self-weight of the structure, fiber-reinforced lightweight aggregate concrete was used in the backfilling works such as sunken toilets.

The following advantages of fiber-reinforced lightweight aggregate concrete are mainly considered:

(1) Light weight

The project is located in the reclamation area. The soil in the reclamation area is relatively soft and the foundation condition is poor. The use of traditional heavy filling materials will increase the self-weight of buildings, thus increasing the risk of foundation settlement. Lightweight aggregate concrete has the characteristics of light weight, which can effectively reduce the structural load of the building, reduce the uneven settlement of the foundation, and improve the stability of the building.

(2) Good thermal insulation performance

The thermal conductivity of lightweight aggregate concrete is small, which can effectively reduce the heat loss, reduce building energy consumption, and improve the comfort of residents in winter. At the same time, it can also avoid the freeze–thaw damage of concrete in winter.

(3) Good crack resistance and impermeability

Fiber can effectively improve the crack resistance of lightweight aggregate concrete and avoid shrinkage cracking of concrete. The toilet is a high-humidity environment, and there is often water vapor and drops. Fiber-reinforced lightweight aggregate concrete can effectively prevent the penetration and absorption of water, keep the interior of the toilet dry, and prevent the breeding of mold.

**Figure 13:**
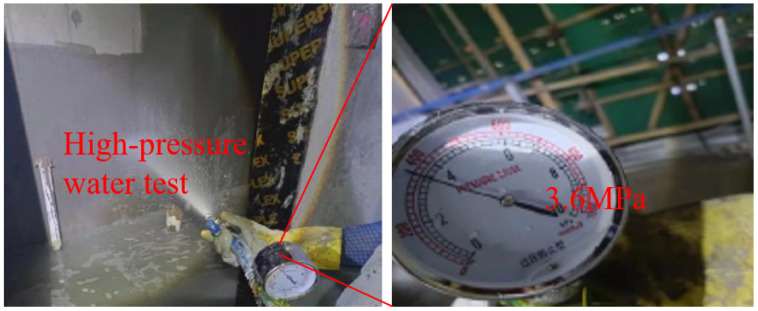
High-pressure water test.

**Figure 14:**
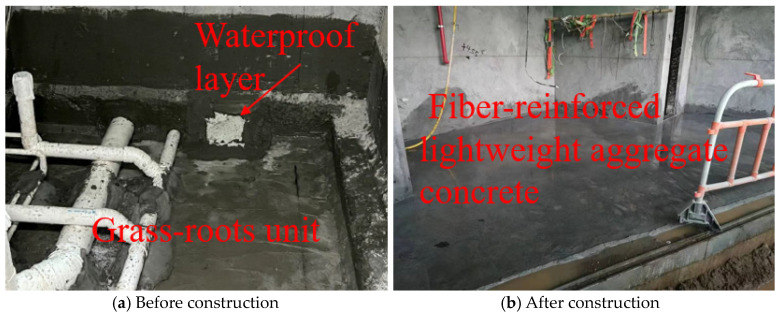
Construction of toilet backfill concrete.

In the backfilling construction of the sunken bathroom, preparatory work was first completed, including cleaning the base layer and preparing materials and equipment for the fiber-reinforced lightweight aggregate concrete. During base layer cleaning, it was ensured that there was no debris or standing water, and waterproofing treatment was conducted if necessary. Next, formwork and partitioning were carried out, with templates set in the construction area to ensure concrete molding quality, and pipeline positions were reserved where needed. The concrete was mixed according to the proportions in Table 2, with fibers added in two stages and mixed thoroughly to ensure an even distribution of fibers. After pouring, it was compacted with a vibrator, with special attention paid to the compactness of corners. After leveling the surface, wet curing was performed, keeping the concrete surface moist for about seven days to ensure adequate strength development. The measured rebound strength of the concrete is shown in Table 4. At 28 days, the strength reached 52.3 MPa, exceeding the design strength. As shown in Figure 13, a high-pressure water test (water pressure greater than 3 bars) was conducted, with each 10 square meters tested for 5 min, and no leakage points were found. As shown in Figure 14, the concrete surface remained intact without cracks, and there was no seepage after the water immersion test.

## 4. Conclusions

(1) A reasonable amount of fiber (6 kg/m^3^) can significantly enhance the splitting tensile strength, flexural strength, and impact toughness of lightweight aggregate concrete, and can significantly improve the damage pattern of concrete. When the fiber content is too small (3 kg/m^3^), it cannot effectively bear the tensile stress generated by compression and has limited restraint on cracks. At the same time, the addition of fibers introduces a weak fiber–matrix interface, resulting in lower strength than fiber-free concrete. When the dosage is too large (9 kg/m^3^), because there are too many fibers, the weak surface of the interface between the fibers and the cement matrix increases, and the fibers are not fully wrapped by the cement slurry, resulting in more defects. This leads to a reduction in the bonding strength between the fibers and the cement paste, and ultimately a reduction in the strength of the concrete. Therefore, it is necessary to set the fiber content reasonably.
(2) During the freeze–thaw cycle, the pore water inside the concrete freezes and generates tensile stress, and tensile cracks are generated inside the concrete. Under the action of repeated freeze–thaw cycles, the cracks gradually accumulate and expand, eventually leading to the destruction of the concrete structure. Freeze–thaw cycles have a great impact on the strength of ordinary lightweight aggregate concrete. After 150 freeze–thaw cycles, the strength loss rate exceeds 25%.
(3) After adding fiber, the fiber bears the tensile stress and hinders the development of internal cracks. After 150 freeze–thaw cycles, the compressive and flexural strength losses of the concrete with 6 kg/m^3^ fiber were reduced by 13.7% and 22.9%, respectively, compared with the concrete without fiber, and the initial cracking energy consumption and failure energy consumption under the impact load are also significantly increased. In the early stages of the freeze–thaw cycle, the strength decreases slowly and the matrix cracks. This part of the load is transferred to the fibers. At this time, the concrete relies on the tensile capacity of the fibers themselves and the friction generated by the fibers and the matrix to simultaneously dissipate energy. This increases the residual strength of the concrete after cracking. At this time, the tensile stress is lower than the tensile strength of the fiber and the fiber–matrix bonding strength, and the fiber absorbs external force through its own deformation ability. However, in the later stages of the freeze–thaw cycle, the tensile stress is greater than the bonding force between the fibers and the matrix, and the fibers will slip or even pull out, losing their gradual toughening effect, resulting in a rapid reduction in the concrete strength.
(4) The influence of freeze–thaw damage on the impact toughness is greater than the compressive strength and flexural strength under static load. Before freeze–thaw, the initial crack energy of the fiber concrete (6 kg/m^3^) is 76% greater than that of ordinary concrete, but after the cycle, it is only 49% greater. This is mainly because the freeze–thaw cycle destroys the bonding strength of the fiber–matrix interface and reduces the ability of the fiber to withstand impact loads. The performance of concrete is influenced by both the fiber content and freeze–thaw cycles.
(5) In this research, the effect of the freeze–thaw cycle on the performance of fiber-reinforced lightweight aggregate concrete under laboratory conditions was studied in detail. Due to the limitations of the experimental conditions and time, the impact of real natural conditions (temperature, humidity, salt exposure) on concrete had not been studied at this stage. Considering that different temperatures, humidity, and other natural environments will have different effects on concrete, the future performance research of lightweight aggregate concrete in a natural environment will be very meaningful.

## Figures and Tables

**Table 1:** Chemical composition of cement (%).

CaO	SiO_2_	Al_2_O_3_	Fe_2_O_3_	MgO	SO_3_	K_2_O	Loss	Total
61.12	22.24	6.40	3.08	2.20	1.88	0.47	1.53	98.92

**Table 2:** Mix proportion of lightweight aggregate concrete (kg/m^3^).

Cement	Sand	Lightweight Aggregate	Water Reducer	Water
440	764	598	6.6	140.8

**Table 3:** Decline rate of mechanical properties after freeze–thaw cycle.

Mechanical Property	Freeze–Thaw	0 kg/m^3^ Fiber	3 kg/m^3^ Fiber	6 kg/m^3^ Fiber	9 kg/m^3^ Fiber
Compressive Strength	50	2.51%	3.72%	1.69%	6.13%
	100	12.71%	12.71%	5.92%	20.35%
	150	20.68%	21.34%	7.02%	24.46%
Tensile Strength	50	3.76%	5.26%	3.38%	9.18%
	100	14.16%	17.17%	9.09%	28.32%
	150	22.25%	26.04%	13.77%	37.24%
Flexural Strength	50	5.83%	4.14%	2.66%	6.23%
	100	22.16%	18.78%	8.47%	24.94%
	150	33.53%	27.62%	10.65%	30.67%

**Table 4:** Measured rebound strength of concrete.

Curing Time	3 Day	7 Day	28 Day
Rebound strength	35.2	46.1	52.3

## Data Availability

The original contributions presented in the study are included in the article, further inquiries can be directed to the corresponding author.
